# NRP1 transduces mechanical stress inhibition via LATS1/YAP in hypertrophic scars

**DOI:** 10.1038/s41420-023-01635-3

**Published:** 2023-09-13

**Authors:** Mengzhi Li, Peng Wang, Jingting Li, Fei Zhou, Shixin Huang, Shaohai Qi, Bin Shu

**Affiliations:** 1 Department of Burns, the First Affiliated Hospital of Sun Yat-sen University, Guangzhou, China; 2 Department of Hand and Foot Surgery, Shandong Provincial Hospital Affiliated to Shandong First Medical University, Jinan, China; 3 Department of Institute of Precision Medicine, the First Affiliated Hospital of Sun Yat-sen University, Guangzhou, China

**Keywords:** Single-molecule biophysics, Genetic testing

## Abstract

Hypertrophic scar (HS) is an abnormal fibrous hyperplasia of the skin caused by excessive tissue repair in response to skin burns and trauma, which restricts physical function and impairs patients’ quality of life. Numerous studies have shown that pressure garment therapy (PGT) is an effective treatment for preventing hypertrophic scars. Herein, we found that mechanical stress stimulates the neuropilin 1 (NRP1) expression through screening GSE165027, GSE137210, and GSE120194 from Gene Expression Omnibus (GEO) database and bioinformatics analysis. We verified this stimulation in the human hypertrophic scar, pressure culture cell model, and rat tail-scar model. Mechanical compression increased LATS1 and pYAP enrichment, thus repressing the expression of YAP. Functionally, the knockdown of NRP1 promoted the expression of LATS1, thus decreasing the expression of YAP and inhibiting endothelial cell proliferation. Furthermore, co-immunoprecipitation analysis confirmed that NRP1 binds to YAP, and mechanical compression disrupted this binding, which resulted in the promotion of YAP relocation to nuclear. In conclusion, our results indicated that NRP1 transduces mechanical force inhibition by inhibiting YAP expression. Mechanical pressure can release YAP bound to NRP1, which explains the phenomenon that mechanical stress increases YAP in the nucleus. Strategies targeting NRP1 may promote compression therapy with optimal and comfortable pressures.

## Introduction

Hypertrophic scar (HS) is a skin abnormal fibrous hyperplasia response to skin burns and trauma, restricting physical function and impairing patients’ quality of life [1]. Despite the rapid advances in prevention and therapy, the morbidity of HS after surgery is still as high as 35% [2]. Pressure garment therapy (PGT) has been used to reduce HS with satisfactory results since 1971 and is still the standard HS strategy today, while the potential mechanism is still unknown [3, 4]. Therefore, elucidating the underlying biological mechanisms of mechanical forces may help to identify reliable biomarkers for HS therapy.

Restricting scar blood flow is considered an essential mechanism of PGT [5]. It revealed that uncontrolled angiogenesis generally promotes pathological processes that spur scarring [6]. Mechanical stress depressed the vascular lumen, potentially favorable to regulate uncontrolled angiogenesis to resistance scar formation. Meanwhile, many discomforts caused by the pressure garments, such as stuffiness, paresthesia, and pressure loss-led replacement, have affected their effectiveness and compliance [7]. Here, we attempt to find the mechanical transducer that transmits the biological function of mechanical stress in vascular endothelial cells.

Several studies have focused on the use of mechanical sensors that transmit mechanobiological influences, such as biological sense systems, responding signals, and control of their mechanical surroundings [8]. Mechanobiological detectors play essential roles in scar formation and are critical for a high success rate of HS compression therapy [9]. However, the specific factors involved in mechanical force transmission and their related mechanisms remain unclear.

Neuropilin 1 (NRP1) is a cell surface transmembrane protein that is expressed in many cell types and acts as a co-receptor for mechanical stimulation [10, 11]. A recent study demonstrated that NRP1 influences the interactions between myofibroblasts and soluble fibronectin, promoting integrin-dependent fibronectin fibril assembly, matrix stiffness, and tumor growth [12]. Whether NRP1 can transmit the biological function of mechanical force is still unknown.

Yes-associated protein (YAP) senses physical properties such as mechanical forces [13]. YAP regulation can either be Hippo pathway-dependent or -independent. Mechanical signals regulate upstream proteins of YAP in the Hippo signaling pathway, like large tumor suppressor kinase 1 (LATS1), affecting the phosphorylation and expression of YAP [14]. Mechanical signal-induced changes in extracellular matrix (ECM) elasticity and cell geometry can directly trigger YAP/TAZ signaling and induce YAP to enter the nucleus [15]. A recent study revealed that blocking mechano-transduction signaling with a YAP inhibitor promotes wound regeneration of secondary skin elements, which has translational implications for scar therapy [16]. YAP is a vital sensor for mechanical microenvironmental changes and a negative wound-healing factor, and it may be involved in PGT inhibition of HS.

In this study, we identified a mechanically sensitive NRP1 positively associated with mechanical force stimulation in vivo and in vitro. We investigated the regulatory role of NRP1 in YAP expression through control of the YAP upstream regulator LATS1. We uncovered that mechanical stress disrupts YAP binding to NRP1, resulting in YAP transportation into the nucleus under mechanical force stimulation. This study demonstrated a novel NRP1 regulatory model of YAP that transduces mechanobiological functions, which might enhance the effects of compression therapy with optimal and comfortable pressure. Our findings provide new insights into the treatment of hypertrophic scars.

## Results

### Mechanical compression increases NRP1 expression in multiple types of human cells

Several human cell types are sensitive to mechanical stress and can translate mechanical signals into biochemical signals due to specific gene mutations and modifications [17]. Therefore, we screened mechanical compression-related genes using data from the Gene Expression Omnibus (GEO) database.

**Fig. 1:**
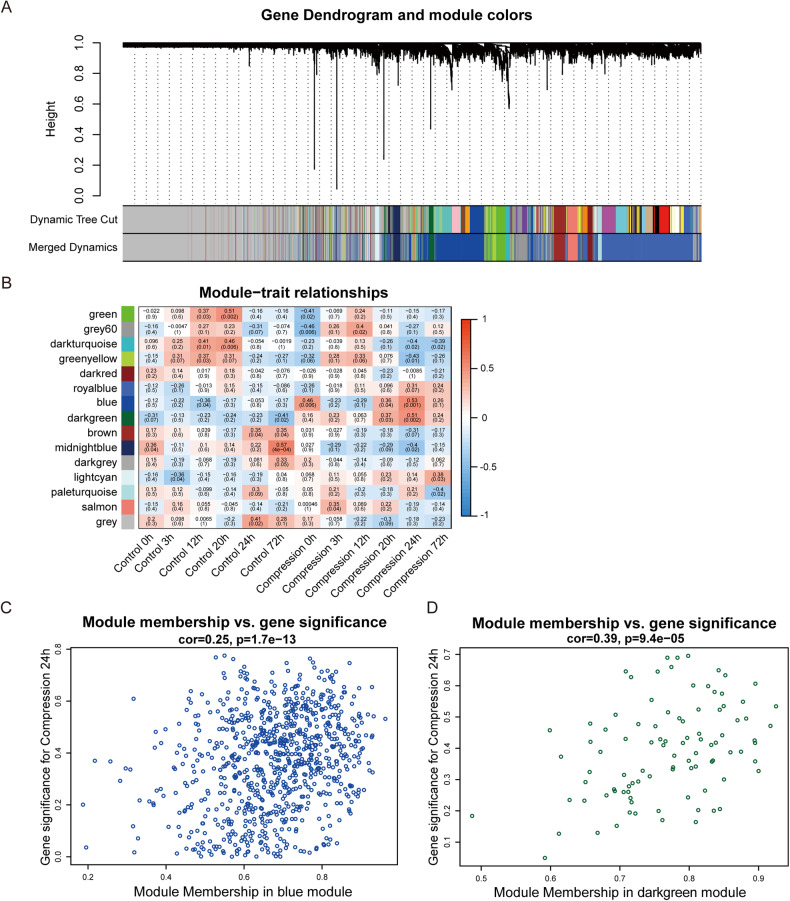
Compression-related-genes identified by WGCNA. **A** Cluster dendrogram of genes clustered based on a dissimilarity measure. **B** Module-trait relationships of module genes that respond to compression. **C**, **D** Scatterplots show the two modules most significantly related to compression in blue and dark green.

First, we retrieved the human transcriptome array including data on the reactions of chondrocytes to mechanical compression (GSE165027) and constructed a co-expression module using weighted gene co-expression network analysis (WGCNA). We selected power of β = 18 (scale-free R^2^ = 0.95) as the soft thresholding parameter to ensure that the network was scale-free. Based on the topological overlap, 15 modules were identified by hierarchical clustering. (Fig. 1A). The modules-trait correlation analysis showed that the top two modules related to compression were the blue (*r* = 0.53, *P* = 0.001) and dark green modules (*r* = 0.51, *P* = 0.002) (Fig. 1B). NRP1 was identified in the dark green module. Correlation analyses between module membership and gene significance in these two modules are shown in Fig. 1C, D.

**Fig. 2:**
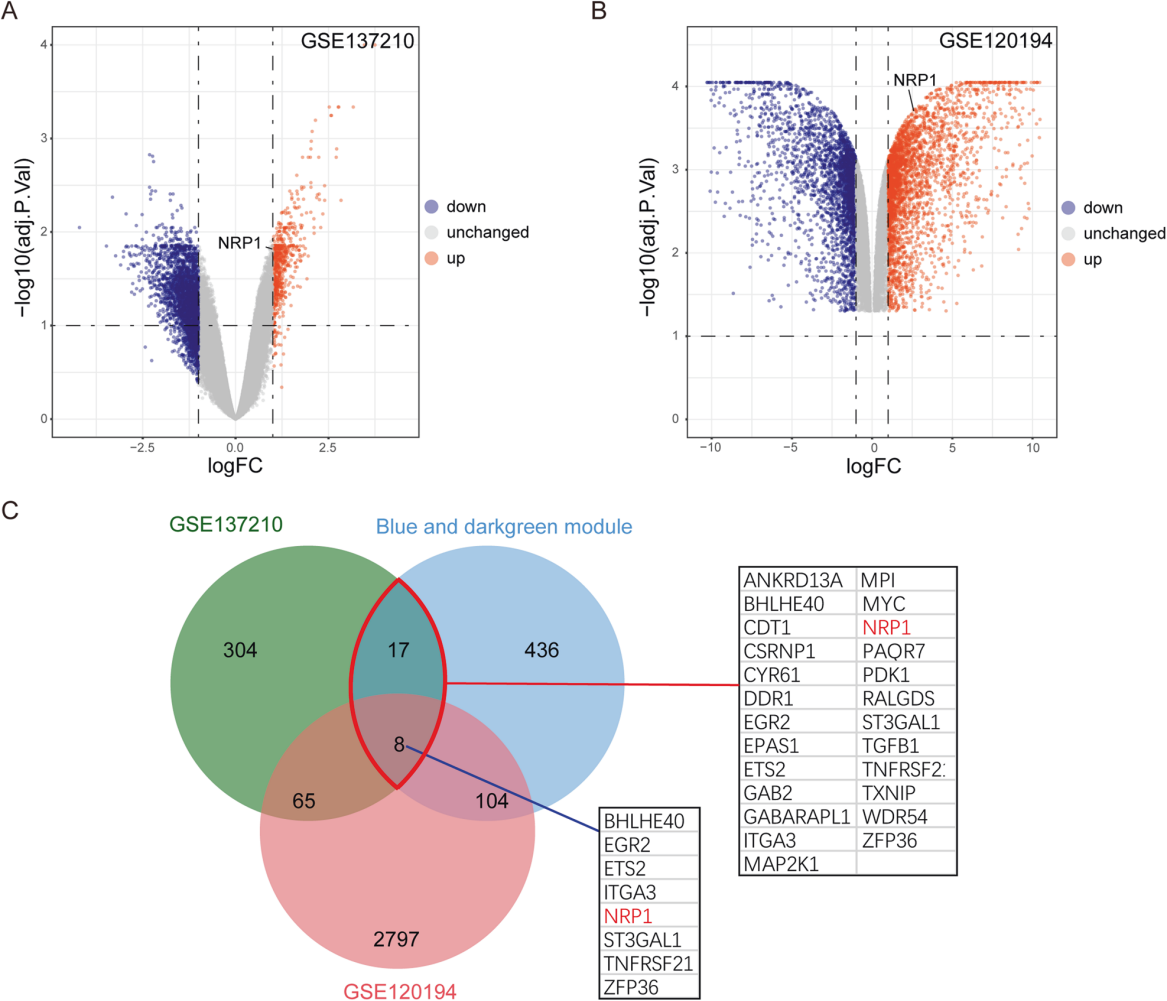
Mechanical compression increases NRP1 expression in multiple types of human cells. **A**, **B** The volcano plot shows upregulated (red) and downregulated (blue) genes, which are identified based on a fold change > 1.0 and a corrected *P*-value < 0.05. NRP1 is included in the upregulated groups. **C** Venn diagram shows the intersection of compression-related modules, GSE137210 upregulated genes, and GSE120194 upregulated genes. NRP1 is included in this intersection.

Next, we verified this result in other GEO datasets. Differentially expressed genes (DEGs) were identified for the upregulated genes to determine the changes in cells under mechanical stress in GSE137210 (glioblastoma cells) and GSE120194 (hepatocellular cells) datasets (Fig. 2A, B). The intersection analysis of these two datasets confirmed that NRP1 was upregulated by mechanical stress in different cell types, as shown in the Venn paragraph diagram (Fig. 2C). These findings suggest that NRP1 expression is associated with mechanical stress in multiple cell types.

### Mechanical compression therapy increases NRP1 expression in dermal endothelial cells of human HS

**Fig. 3:**
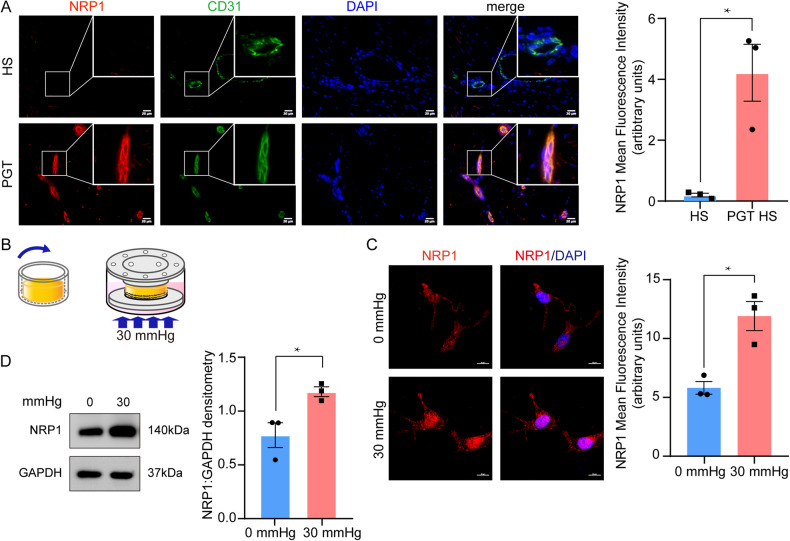
Mechanical therapy increases NRP1 expression in hypertrophic scar dermal endothelial cells. **A** Fluorescent multiplex immunohistochemical staining on hypertrophic scar tissues (*n* = 3), PGT treated hypertrophic scar (*n* = 4). These results indicate NRP1 was selectively secreted in endothelial cells. **B** The process of the development of a 3D cell-cultured hydrogel model. **C** Immunofluorescence staining show remarkably increased NRP1 expression in response to a 30 mmHg mechanical force. **D** Western blot results show that 30 mmHg compression significantly promotes NRP1 expression. The results are expressed as the means with SEM (*n* = 3). A two-tailed Student’s t-test was used for all analysis. *P < 0.05.

PGT inhibits HS formation by decreasing blood flow [5] however, this process is poorly understood. Therefore, we stained four HS tissues that received PGT and required a secondary operation to detect NRP1. CD31 staining revealed that NRP1 was selectively located in HS endothelial cells (Fig. 3A). Collectively, these results indicated that NRP1 is selectively located on the dermal endothelial cell membrane, and mechanical stress promoted NRP1 expression in human HSs.

NRP1 is a sensory co-receptor of intercellular forces [18] and shear stress [11]. We constructed a 3D hydrogel model to investigate whether mechanical force promotes NRP1 expression in human dermal endothelial cells (Fig. 3B). Immunofluorescence staining of hydrogel cells showed that NRP1 expression increased significantly in response to 30 mmHg compression (Fig. 3C), which was consistent with the immunoblotting results (Fig. 3D).

### NRP1 inhibition impairs sensitivity to mechanically induced anti-angiogenesis in vitro

**Fig. 4:**
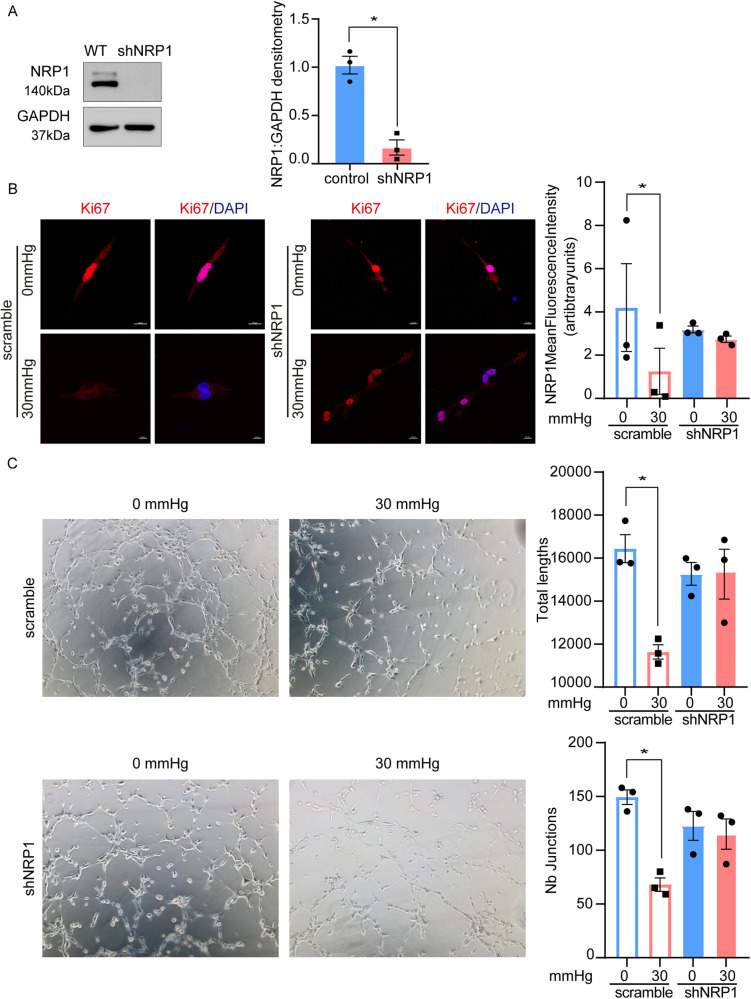
NRP1 inhibition impairs the sensitivity to mechanically induced anti-angiogenesis in vitro. **A** Western blot was used to validate and select NRP1 shRNA lentiviral vectors. **B** Immunostaining results demonstrate that Ki67 decreased after mechanical stimulation. In contrast, NRP1-deficient cells showed no difference in Ki67 immunostaining under 30 mmHg force or 0 mmHg force. **C** NRP1-deficient cells did not show a noticeable difference in response to 0 mmHg or 30 mmHg stress inhibition in the complementary tube formation assay. The results are expressed as the means with SEM (*n* = 3). A two-tailed Student’s t-test was used for all analysis. *P < 0.05.

To determine whether NRP1 transduces mechanical forces in HDMECs, we used NRP1-shRNA to knockdown NRP1 in HDMECs and evaluated proliferation and tube formation in the control and NRP1-deficient cells. NRP1 knockdown was confirmed using western blotting (Fig. 4A). Immunostaining of Ki67 demonstrated that mechanical force inhibited Ki67 expression. NRP1-deficient cells expressed lower levels of Ki67 and showed no difference in Ki67 expression with or without mechanical forces (Fig. 4B). Similar results were obtained in the tube formation assay. The results of the mechanical inhibition of tube formation assay demonstrated no significant difference in the Nb junctions and branches in NPR1-deficient cells. (Fig. 4C) Furthermore, NRP1 knockdown partially rescued the anti-angiogenic effects induced by mechanical forces. These findings suggest that NRP1 contributes to the mechanical-stress response in dermal endothelial vascular cells.

### Mechanical stress increases NRP1 expression in rat tail-scar

**Fig. 5:**
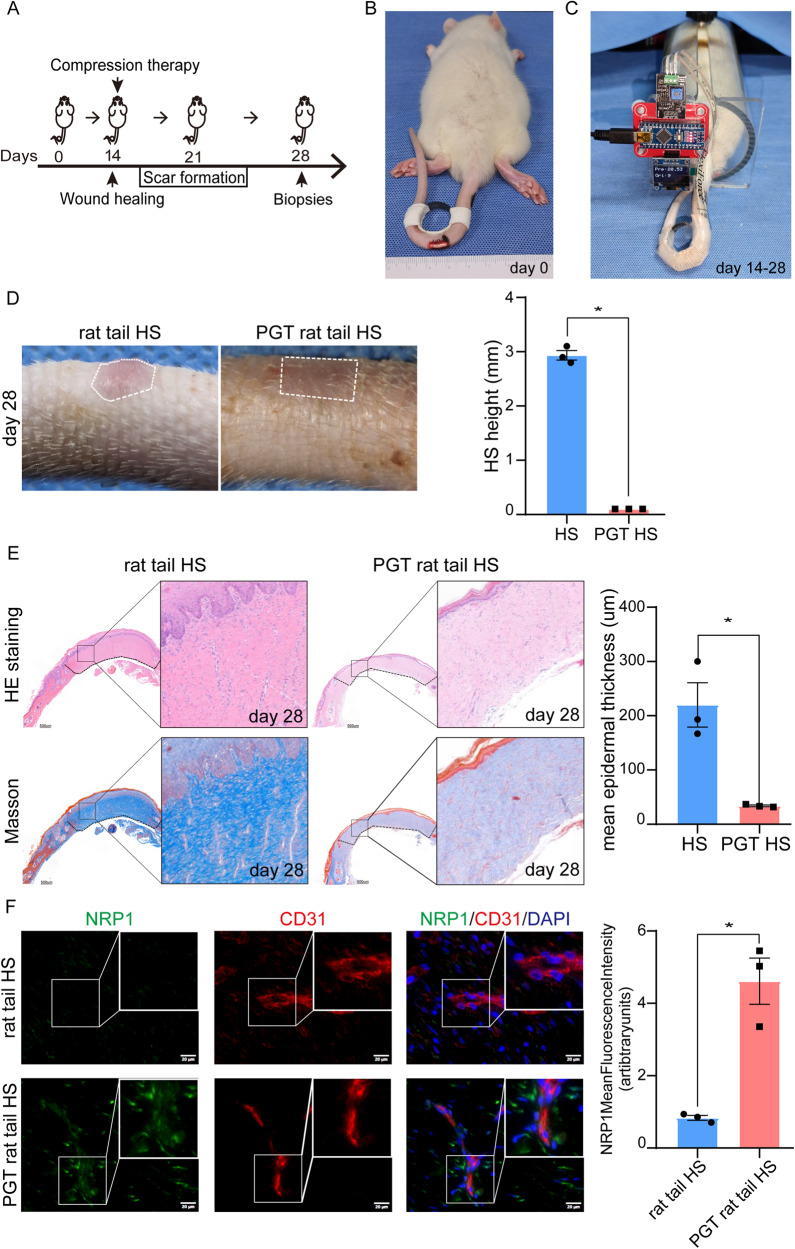
NRP1 expression is increased by mechanical stress in rat tail-scar. **A** Pressure garment inhibits rat tail-scar setup. **B** A 2 cm diameter iron ring was used to induce a stretchable hypertrophic scar in the rat tail. **C** A pressure garment was applied with constant pressure to inhibit rat tail-scars. **D** The morphological images demonstrate that the hypertrophic scar was reduced through compression treatment. **E** The histological images reveal that pressure garment-treated scars exhibited reduced collagen deposition and the presence of parallel collagen bundles within the epidermis. Scars treated with pressure garments also showed a thinner mean epidermal layer. **F** Fluorescent multiplex immunohistochemical analysis shows that mechanical compression significantly promotes NRP1 expression in rat tail model and co-location of NRP1 and CD31. The results are expressed as the means with SEM (*n* = 3). A two-tailed Student’s t-test was used for all analysis. *P < 0.05.

A novel and reliable rat tail model with biological features similar to those of human HSs has been developed and used for morphological and molecular studies [1, 19]. To validate the relationship between NRP1 and mechanical forces in vitro, we used the HS rat tail model and applied a compression garment to the rat tail as shown in Fig. 5A–C. Thirty rats were randomly divided into two groups according to whether they received PGT. First, we induced a 6 × 6 mm full-thickness skin wound on each tail and stretched it with a 2 cm steel ring. After 14 days, most of the wounds had healed. We treated the wound with a pressure garment in the compression therapy group. Approximately 21 days later, scar formation was apparent in the control group. All tail-scar specimens were harvested at 28 days. Morphological and histological analyses showed that hypertrophic scarring was successfully induced on day 28 and was reduced by compression garment treatment. The compression garment-treated scars displayed decreased epidermal thickness, less collagen deposition, reduced blood and parallel collagen bundles in the epidermis (Fig. 5D, E). Moreover, immunofluorescence analysis demonstrated that mechanical compression significantly promoted NRP1 expression in rat tail-scar dermal endothelial cells (Fig. 5F). These findings indicated that mechanical compression induces NRP1 expression inhypertrophic scar rat model tails.

### NRP1 inhibition reduces the mechanical force-induced through YAP mediated pathways

**Fig. 6:**
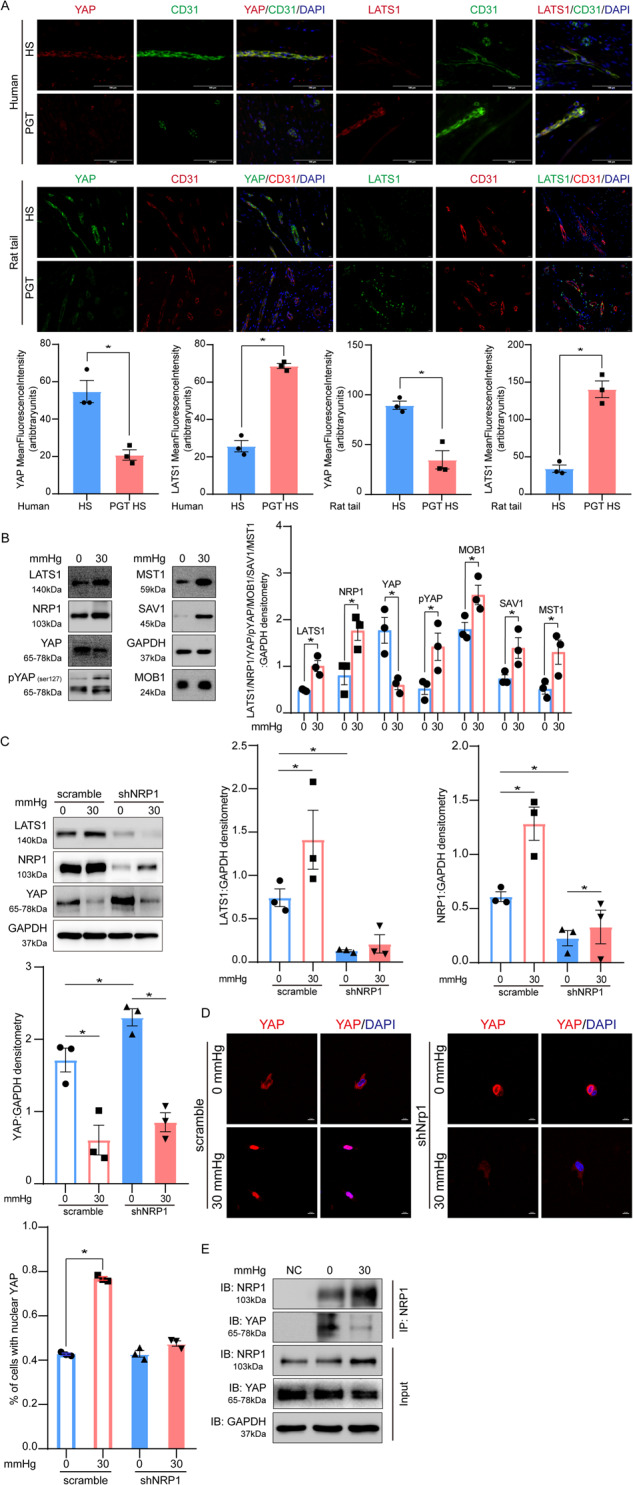
NRP1 inhibition reduces mechanically induced YAP expression, YAP binding to NRP1, and YAP nuclear-cytoplasmic ratio. **A** Fluorescent immunohistochemical results show that compression therapy significantly reduced YAP and increased LATS1 expression in human hypertrophic scars and rat tail hypertrophic scars. **B** Immunoblotting analysis reveals concurrent increases in LATS1, NRP1, pYAP (ser127), MST1, SAV1, and MOB1 expression and a reduction in YAP expression. **C** Immunoblotting results show that NPR1 knockdown abolished stimulation of LATS1, promoting YAP expression. When subjected to 30 mmHg, NRP1 knockdown prevented LATS1 promotion and YAP inhibition. **D** Immunofluorescence results show that the mechanical force increased YAP nuclear location in the 3D cultured HDMECs hydrogel. NRP1 knockdown counteracts the increased nuclear/cytosolic YAP ratio under high mechanical stress. **E** Co-immunoprecipitation shows that NRP1 co-precipitated with YAP without stimulation. However, mechanical compression inhibited this binding despite increased NRP1 expression. The results are expressed as the means with SEM (*n* = 3). One-way ANOVA was used for all analysis. *P < 0.05.

Our immunostaining results showed that compression therapy significantly reduced YAP expression and promoted LATS1 expression in both human hypertrophic scars and rat tail scars tissues (Fig. 6A). These findings suggest a novel mechanism of compression therapy for the treatment of hypertrophic scars.

Since we observed that mechanical forces significantly inhibited YAP expression, we had to investigate the relationship between NRP1, YAP and LATS1. Immunoblotting analysis revealed a concurrent increase of LATS1, MST1, Salvador family WW domain-containing protein 1 (SAV1), and Mps one binder 1 (MOB1) expression, while a reduction in YAP expression under 30 mmHg compression (Fig. 6B).

LATS1-dependent signaling is widely recognized as a regulator of YAP inhibition and cytoplasmic localization [20]. However, the mechanism of this mechano-transduction is unclear [21, 22]. We speculated that NRP1 regulates YAP inhibition via LATS1. Consistent with this notion, NRP1 knockdown abolished LATS1 stimulation, which promoted YAP expression. When subjected to an increasing mechanical force, NRP1 knockdown prevented the increase in LATS1 and decrease in YAP. NRP1 mediates the interference of mechanical stress on YAP through LATS1, but mechanical stress is another essential inhibitor of YAP expression. (Fig. 6C).

**Fig. 7:**
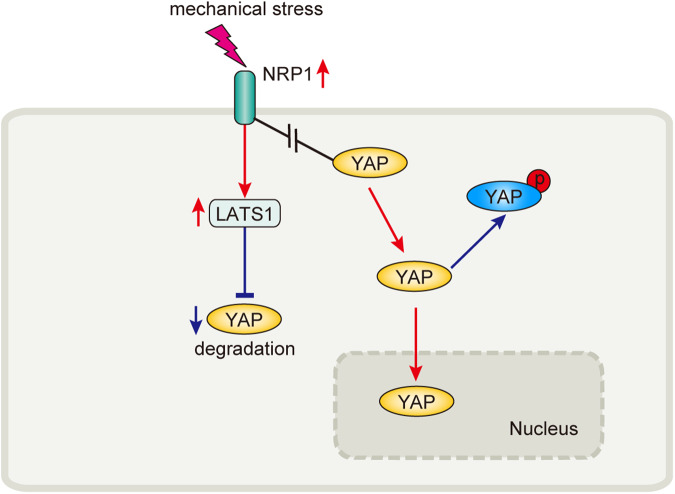
YAP regulation by NRP1 in response to a mechanical force stimulus. NRP1 inhibits YAP expression through increasing LATS1 expression; Mechanical stress prevents the binding of NRP1 and YAP.

Additionally, we evaluated the effects of mechanical compression on the upstream YAP regulators at various mechanical strengths (0 mmHg and 30 mmHg) matching the pressure of clinical PGT [5] for 24 h. Immunofluorescence staining showed that the mechanical force increased YAP nuclear localization in the 3D cultured HDMECs hydrogel. NRP1 knockdown counteracted the increased nuclear/cytosolic YAP ratio under high mechanical stress (Fig. 6D). Notably, YAP activity was also affected by mechanical signals in a Hippo pathway-independent manner [20]. To investigate whether NRP1 binds to YAP and whether mechanical cues influence this binding, we used co-immunoprecipitation to detect binding between NRP1 and YAP at 0 and 30 mmHg compression. NRP1 co-precipitated with YAP without stimulation; however, this binding was inhibited by mechanical compression despite increased NRP1 expression (Fig. 6E). These findings indicated that mechanical compression triggered YAP entry into the nucleus after inhibiting the binding between YAP and NRP1. The released YAP then translocates into the nucleus. These results elucidate a novel regulation of mechanical stress in which NRP1 binds to YAP and inhibits YAP expression (Fig. 7).

## Discussion

Pressure clothing therapy (PGT) is the current standard for preventing and treating post-burn scars [23]. It works by inhibiting blood flow and endothelial cell function, which can help to reduce scar thickness [4]. After undergoing PGT treatment, the number of patients requiring surgical intervention is relatively low. However, the transmission mechanism between the mechanical press and biological outcomes remains unknown [24]. Here, we attempted to find a mechanical transducer by which mechanical stress transmits its biological functions and inhibits HS formation. We used bioinformatics analysis and three mechanical compression-related GEO databases to reveal that mechanical force promotes NPR1 expression in multiple human cell types. Furthermore, we demonstrate that mechanical compression inhibits angiogenesis in hypertrophic scar through NRP1-LATS1-YAP interactions in vivo and in vitro.

NRP1 is a mechanoreceptor on the endothelial cell membrane that can regulate atherosclerotic plaque formation by sensing shear stress resulting from blood flow [10, 11]. It is clear that NRP1 plays an essential role in inhibiting angiogenesis [25], which is a critical process in wound repair [26]. Since PGT promotes NRP1 expression in hypertrophic scar endothelial cells, we used endothelial cells to detect whether NRP1 transmits the biological function of mechanical compression.

The Flexercell FX-5000 compression system is a recognized cell pressure culture system for biomechanical effects studies [27]. In this study, we adjusted the composition ratio and increased the gel column size for pressure culture. We cast collagen in a 24-well cell culture plate and then transferred it to the press plate. These attempts ensure that more cells can live in the gel and that the volume of the glued column is consistent. We revealed that mechanical stress inhibited HDMECs proliferation and tube-forming ability and upregulated NRP1 expression. Furthermore, knockdown NRP1 expression restrained the inhibition of mechanical stress on the proliferation and tube-forming ability of HDMECs. These results suggest that NRP1 is a transducer of mechanical force.

To explore the downstream mechanism that NRP1 mediates mechanobiological effect, we analyzed the effects of the inhibition of NRP1 on YAP and Hippo signaling pathways. It is widely believed that LATS1 is the upstream regulator of YAP and enhances YAP phosphorylation to inhibit its transportation to the nucleus in response to mechanical signals [20]. Meanwhile, mechanical force is also the direct activator of the YAP nuclear localization [13]. In this study, we revealed that mechanical stress promoted LATS1 and inhibited YAP in human and rat tail HS models. Mechanical pressure inhibited YAP expression and promoted pYAP and LATS1 expression, consistent with the principle of inhibiting YAP and promoting scar-free healing confirmed by a previous study [16]. These results highlight the clinical value of the LATS1/YAP signaling axis in mechanical pressure-induced scar hyperplasia inhibition. We also confirmed that culturing HDEMCs at 30 mmHg pressure for 24 h increased LATS1 expression and decreased YAP expression. NRP1 knockdown affects YAP activity is plausible because NRP1 regulates LATS1. At the same time, although the expression of YAP was significantly increased after the inhibition of NRP1, the inhibitory effect of mechanical pressure on YAP was still significant. These suggest that NRP1 mediates the interference of mechanical stress on YAP, but mechanical stress is another essential inhibitor of YAP expression.

The current view implicates mechanical force as a direct activator of the YAP nuclear localization [13]. Since YAP is an essential sensor of the cellular mechanical microenvironment, we investigated the interactions between NRP1 and YAP. Co-immunoprecipitation indicated that NRP1 binds to YAP and that mechanical compression disrupted this binding. These results suggested that mechanical stress weakens the binding of NRP1 to YAP, which can explain the entry of YAP into the nucleus.

In conclusion, we identified a gene, NRP1, that is related to response to mechanical signals, using online databases, clinical hypertrophic scar specimens, pressure-cultured HDMECs models, and pressure-inhibited rat tail-scar models. Mechanosensitive transmembrane NRP1 mediates the biological effects of mechanical stress through the LATS1/YAP signaling axis. NRP1 mediates the interference of mechanical stress on YAP, but mechanical stress is another essential inhibitor of YAP expression. Therefore, targeting NRP1 may promote compression therapy with optimal and comfortable pressures. Our findings can help improve the curative effect of PGT and the quality of life for patients with HS.

## Materials and methods

### Fluorescent multiplex immunohistochemical analysis of human samples

This study was approved by the institutional review board of the First Affiliated Hospital of Sun Yat-sen University, China. This study observed seven patients with pathological scars who required surgery. Four of the patients were male and had been scalded by hot water. Their ages ranged from 5 to 11 years old, and the time since their injuries ranged from one year to nine years. The hyperplastic scars were located on various parts of their bodies, including the right forearm, left upper limb, right upper limb, and left thigh. All four male patients had been treated with PGT for at least nine months. Before surgery, the Vancouver Scar Scale (VSS) scores were 12, 5, 6, and 10. In addition to these male patients, there was also a seven-year-old girl with contracture scar tissue on her right foot from a hot water burn six years prior (VSS: 10). The other two cases of hyperplastic scar tissue came from a 12-year-old boy who had suffered from hot water burns on his face and neck (VSS: 11) for over ten years. A 4-year-old girl with hot water burns on her neck (VSS: 8) for three years.

Representative FFPE sections were prepared for epitopes available for antibody binding, including dehydration, deparaffinization, and antigen retrieval, after which endogenous peroxidases were quenched before antibody staining. The sections were fixed in 4% paraformaldehyde for 30 min, washed thrice with PBS, and incubated with 0.01% (v/v) Triton X-100 in 5% bovine serum albumin (BSA) at 37 °C for 30 min. Slides were incubated with anti-rabbit NRP1 (1:100, ab81321, AB_1640739, Abcam, Cambridge, UK) at 4 °C overnight. The SignalStain® Boost Detection Reagent (HRP rabbit, #8114) was equilibrated to 37 °C for 60 min. The antibody solution was removed and the sections were washed thrice with TBST for 5 min each. Subsequent experimental steps were performed in the dark. One-hundred microliter Opal 570 Reagent (1:100, Akoya Biosciences, Marlborough, MA, USA) was added per slide and incubated for 10 min at 37 °C in a humidified chamber. The slides were then washed thrice with TBST (5 min per wash).

Subsequently, the antigen unmasking step was repeated for stripping. Using a microwave, the slides were heated in 10 mM sodium citrate buffer (PH 6.0) at sub-boiling temperature for 10 min and then cooled to 37 °C for 30 min. The slides were then incubated with anti-LATS1 (1:100, ab234820, Abcam, Cambridge, UK), anti-rabbit YAP (D8H1X) (14074, Cell Signaling Technology, 1:100, Cambridge, MA, USA), and anti-rabbit CD31 antibodies (1:100, 11265-1-AP, Proteintech Group, China) overnight at 4 °C. The SignalStain® Boost Detection Reagent (HRP rabbit, #8114) was equilibrated, sections were washed, 100 µl Opal 520 Reagent (1:100, Akoya Biosciences, Marlborough, MA, USA) was applied, and then washed thrice with TBST. All slides were counterstained with DAPI for 5 min and images were captured using an epifluorescence microscope (BX63, Olympus, Japan).

### Cell compression model

Human dermal microvascular endothelial cells (HDMECs), purchased from ScienCell Research Laboratories Inc. (Carlsbad, CA, USA), were cultured in Dulbecco’s modified Eagle’s medium nutrient mixture F-12 (DMEM/F-12, Gibco, Waltham, MA, USA) supplemented with 10% FBS. For the 3D cell culture hydrogels, various ingredients were mixed in one well of a 24-well cell culture plate. A mixture of 18 µl NaOH (0.1 mg) and 42 µl 10 × PBS was added to 300 µl type I collagen from rat tail tendon (Xinyou Biotechnology Co., Ltd. China) and then mixed with 1 mL DMEM/F-12 with 10% FBS and 2 × 10^6^ HDMECs suspension after neutralization. After 6–8 h of incubation, each gel was transferred to a well of a BioPress Compression Plate. The pressure force was set at 0.12 and 0.20 at 1 Hz frequency (static wave). For the control, the gels were transferred but unloaded. HDMECs were isolated from these hydrogels using type I collagenase (SCR103, Sigma, Darmstadt, Germany) at a 1 mg/mL dilution for subsequent experiments.

### Tube formation assay

In the tube formation assays, cells were seeded in μ-Slide Angiogenesis dish (#81507, Ibidi, Munich, Germany). Briefly, 10 µl Matrigel (#356231, Corning, Corning, USA) was added to each well and incubated for 1 h at 37 °C. Compression-treated or other treated HDMECs were resuspended in 50 µl F12-DMEM with 1% FBS and seeded in the wells at a density of 5000 cells. The slides were incubated at 37 °C in 5% CO_2_ for 4 h. Tube formation was captured using an inverted epifluorescence microscope (IX83, Olympus, Japan) and analyzed by measuring the total tube length and nodes by ImageJ (National Institutes of Health, Bethesda, MD, http://rsb.info.nih.gov/ij/).

### shRNA lentiviral particle transduction

Human NRP1 short hairpin RNA (shRNA) lentiviral vectors were purchased from VectorBuilder. The IDs used were VB900072-0600gbe (pLV-shRNA#1-Puro), VB900072-0593myd (pLV-shRNA#2-Puro), and VB900072-0589fxv (pLV-shRNA#3Puro). The sequences of the sense oligonucleotides were CAGCCTTGAATGCACTTATAT (shRNA#1), TAAATGTGGCGATACTATAAA (shRNA#2), and TGTGGATGACATTAGTATTAA (shRNA#3). HDMECs were grown to 30–40% density and co-transfected with polybrene (5 µg/mL) and pNRP1 vectors. Western blotting was used to validate the transfection efficiency of shRNA#3 for subsequent experiments.

### Pressure garment application to a scar model generated in rat tail

We followed the stretch-induced rat tail scarring module designed by the Shanghai Ninth People’s Hospital [19]. The Animal Care and Use Committee of the First Affiliated Hospital of Sun Yat-sen University approved all procedures. To validate the NRP1 responses to compression therapy, we selected eight-week-old male Sprague-Dawley rats and induced a 6 × 6 mm full-thickness skin wound on the top of their tails, 4.5 cm from the root. After two weeks, most of the wounds had healed. We treated the wounds with a pressure garment in the compression therapy group. The pressure applied on rat tail is 9.35 mmHg (20 ibs). Both groups were stretched persistently until the end of the experiment. Next, we stained the tail rat sections with immunohistochemical analysis, hematoxylin-eosin staining, or Masson’s trichrome staining.

Rat tail pressure garments were designed and made by the Rehabilitation Department of the First Affiliated Hospital of Sun Yat-sen University. We used a densely woven nylon elastane material to fabricate the garments. We applied a FlexiForce Sensor system (Tekscan, USA) to ensure that the pressure applied on the wounds was consistent.

### Weighted gene co-expression network analysis (WGCNA)

To explore the genetic change in response to mechanical compression, we selected GSE165027, which includes the responses of chondrocytes to mechanical compression. We then constructed a co-expression network using the WGCNA algorithm with the R package [28, 29]. After cluster analysis and power value set, we created a scale-free network. Module eigengenes (MEs) were used to estimate the correlation between the module and different compression time gradient features. Gene significance (GS) represented the mediator *P*-value for each gene to define the correlation between gene expression and compression time gradients. We selected the top two most significant modules related to mechanical compression according to *P* < 0.01 and the higher GS value. NRP1 was included in this module.

### Differential expression (DEGs) analysis

We selected GSE120194 and GSE137210 datasets, which include the prominent features in response to mechanical compression, from the Gene Expression Omnibus (GEO) database. GSE120194 included differentially expressed mechanically responsive mRNAs in human hepatocellular carcinoma cell lines. GSE137210 compared gene expression changes in glioblastoma cells (LN229) treated with 23 Pa compressive solid stress (CSS) or no CSS. The data were normalized via log2 transformation for analysis. Data filtering, original annotation, quality control, normalization, and DEG identification were performed in RStudio (version 1.2.5042) using the GEOquery, limma, stringr, and ggplot2 packages. The thresholds were |logFC | > 1 and *P* < 0.05. The Venn plot displays the common genes after mechanical compression stimulation in three different human sources.

### Gene function prediction

GeneMANIA Cytoscape plugin [30] was used to construct a gene regulatory network using 25 intersection genes. These networks contained co-localization, physical interactions, and genetic interaction networks based on the calculated gene list-specific weights, including kinases, transcription factors, enzymes, and gene regulatory relationships.

### Western blot analysis

Total protein was extracted using radioimmunoprecipitation assay (RIPA) buffer (Invitrogen, Waltham, MA, USA) containing a protease inhibitor cocktail and phosphatase inhibitor cocktail (1:100; #5871 and #5870, respectively; Cell Signaling Technology, Danvers, MA, USA). Briefly, 20 µl of each sample was loaded onto sodium dodecyl sulfate-polyacrylamide gel electrophoresis (SDS-PAGE). The membranes were blocked with 5% BSA for 1 h at 37 °C. Membranes were incubated with the following primary antibodies overnight at 4 °C: NRP1 (1:1000, ab81321, Abcam, Cambridge, UK) and the Hippo Signaling Antibody Sampler Kit (1:1000, #8579, Cell Signaling Technology, Danvers, MA, USA). Horseradish peroxidase (HRP)-linked secondary antibody was incubated with the membranes. Immobilon Western HRP Substrate (Merck KGaA, Darmstadt, Germany) was used to detect protein bands. Its intensity was quantified by densitometry using the ImageJ software (National Institutes of Health, Bethesda, MD, USA).

### Immunofluorescence

The distribution and localization patterns of NRP1, LATS1, YAP, and Ki67 in HDMECs cultured in 3D cell culture hydrogels were detected using immunofluorescence. The gels were fixed in 4% paraformaldehyde for 30 min, washed thrice with PBS, and incubated with 0.2% (v/v) Triton X-100 in 5% BSA at 37 °C for 1 h. The hydrogels were incubated with anti-rabbit NRP1 (1:100, ab81321, Abcam, Cambridge, UK), anti-rabbit LATS1 (1:100, bs-7913R, Bioss Antibodies, China), anti-rabbit YAP (D8H1X) (14074, Cell Signaling Technology, 1:100, Danvers, MA, USA), anti-rabbit Ki67 (1:100, 14-5698-82, RRID: AB_10854564, Thermo Fisher Scientific, Waltham, MA, USA) at 4 °C overnight, washed thrice with PBS, and incubated with Alexa Fluor 555- and 488-conjugated goat anti-rabbit secondary antibodies (1:500; P0179 and P0176, respectively; Beyotime Institute of Biotechnology, Shanghai, China) at 37 °C for 1 h in the dark. All sections were counterstained with 4′,6-diamidino-2-phenylindole (DAPI) (1:1000, C1005, Beyotime Institute of Biotechnology, Shanghai, China) for 5 min. Images were captured using a Zeiss LSM 880 confocal microscope and processed using LSM image software.

### Statistical analyses

SPSS 22 (SPSS Inc., Chicago, IL, USA) was used for statistical analyses. Graph the statistical results using GraphPad Prism 8 (GraphPad Software Inc., San Diego, CA, USA). A two-tailed Student’s t-test was used for comparisons between two groups. One-way ANOVA was used for comparing multiple groups. *P* < 0.05 was considered to indicate a significant difference. At least three independent replicates were used for each experiment. The results are expressed as the means ± standard error of mean (SEM).

### Supplementary information

Original Data File

## Data Availability

All data are available in the main text or the supplementary materials.
