# Supplementary figures and images for: NRP1 transduces mechanical stress inhibition via LATS1/YAP in hypertrophic scars
Source: Cell Death Discov. 2023 Sep 13;9:341. doi: 10.1038/s41420-023-01635-3 (PMC10499927; doi:10.1038/s41420-023-01635-3)

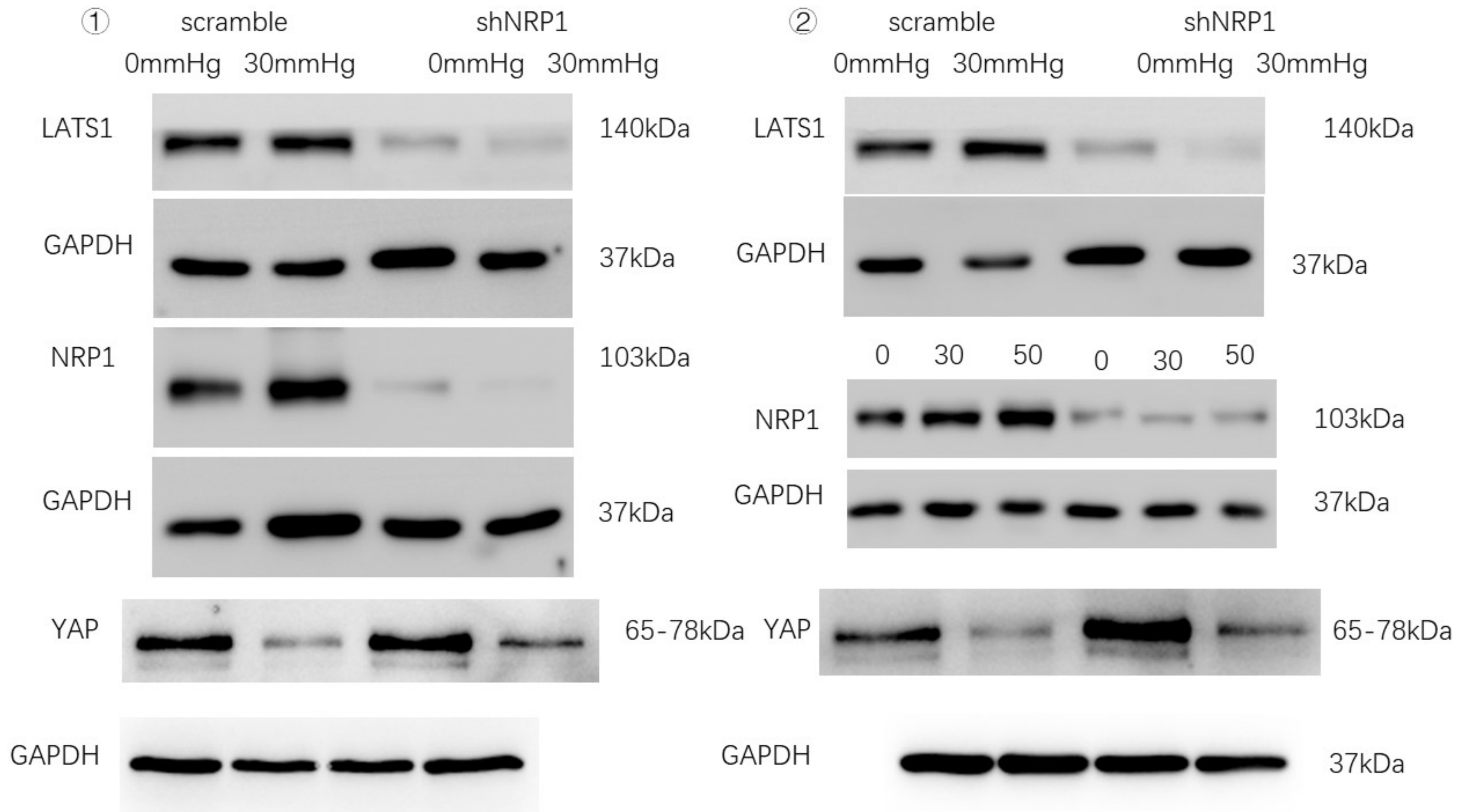

③

scramble

shNRP1

0mmHg 30mmHg

0mmHg 30mmHg

LATS1

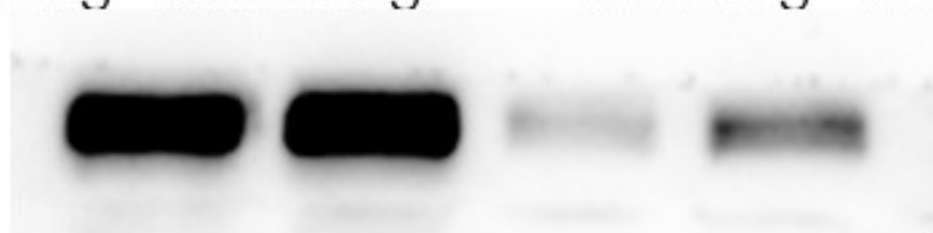

140kDa

GAPDH

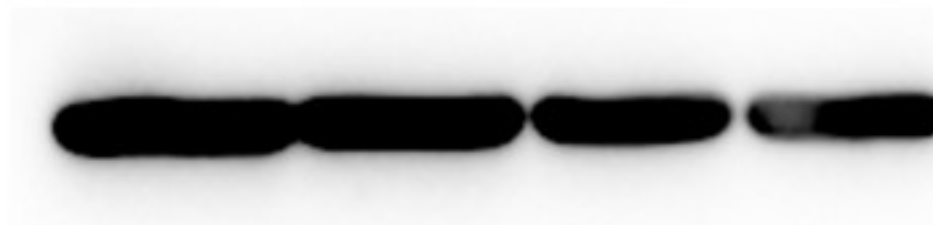

37kDa

YAP

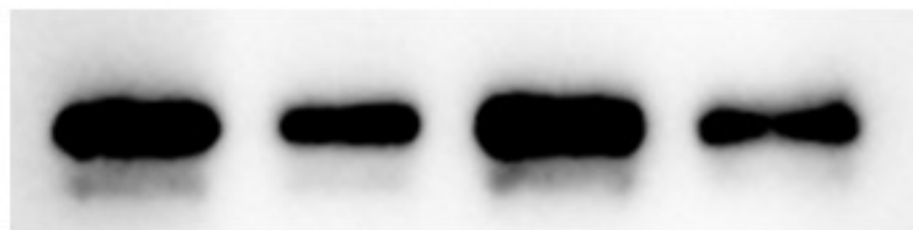

65-78kDa

GAPDH

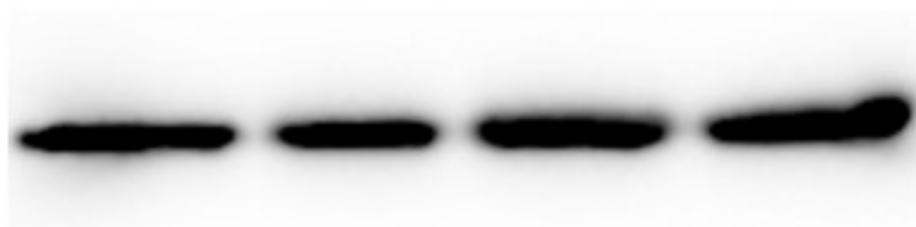

37kDa

NRP1

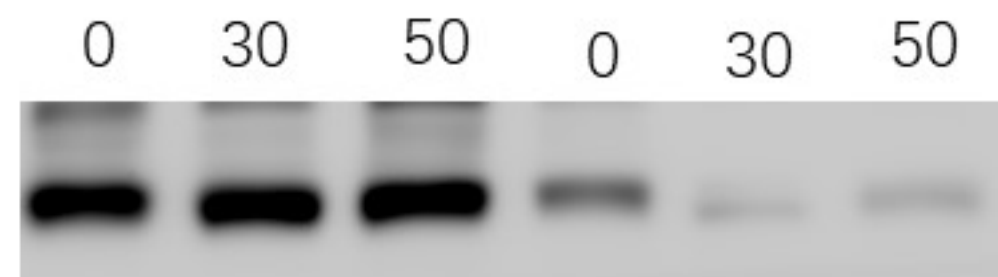

103kDa

GAPDH

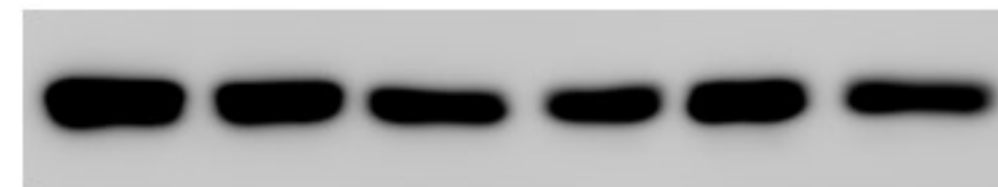

37kDa

— — —

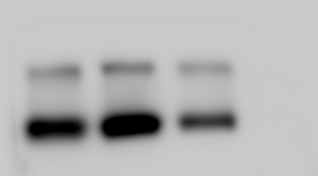

— —

11



— — —

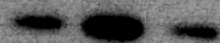

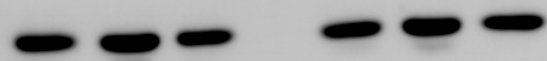

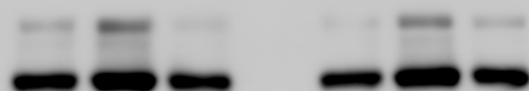

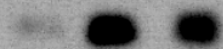

---

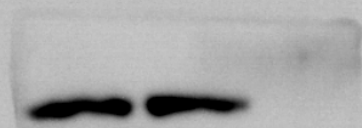

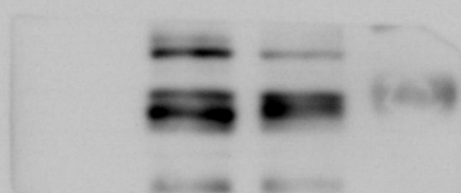

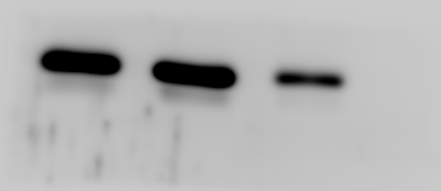

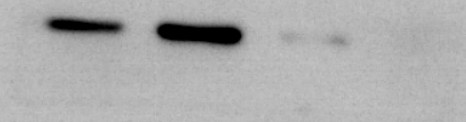

— — —

100

— — —

— — —

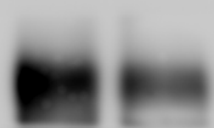

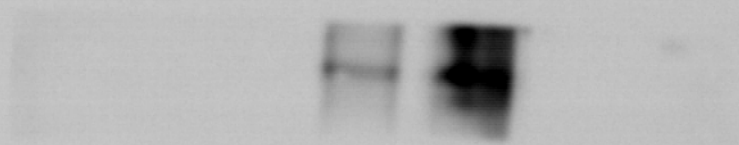

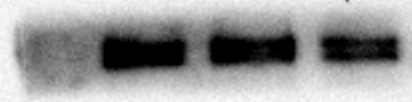

Supplement: Supplementary file 1 — Original Data File [file 41420_2023_1635_MOESM1_ESM.pdf]
